# Psychological consequences of prolonged shelter residence after the Kahramanmaraş earthquakes: a mixed-methods study

**DOI:** 10.3389/fpubh.2026.1787914

**Published:** 2026-05-12

**Authors:** Metin Çınaroğlu, Eda Yılmazer

**Affiliations:** 1 Department of Psychology, Faculty of Administrative and Social Science, Istanbul Nişantaşi University, Istanbul, Türkiye; 2 Department of Psychology, Faculty of Social Science, Beykoz University, Istanbul, Türkiye

**Keywords:** environmental stress, family functioning, internalizing symptoms, Kahramanmaraş earthquakes, prolonged displacement

## Abstract

**Background:**

Prolonged displacement following natural disasters poses significant risks to mental health, yet limited research has examined the chronic psychological consequences of long-term residence in temporary shelters. Two years after the 2023 Kahramanmaraş earthquakes, hundreds of thousands of survivors in Türkiye continue to live in container settlements, providing a critical context for examining sustained perceived stress and family dynamics.

**Methods:**

This convergent mixed-methods study investigated the relationships between perceived stress, family functioning, and internalizing symptoms among adult earthquake survivors residing in temporary shelters. Quantitative data were collected from 413 participants using the Perceived Stress Scale, Family Assessment Device, and Brief Symptom Inventory. A regression-based mediation analysis with bootstrapping (PROCESS Model 4) was conducted to test whether family functioning mediated the association between perceived stress and psychological distress. In parallel, semi-structured qualitative interviews were conducted with a purposive subsample of 41 participants and analyzed using thematic analysis.

**Results:**

Higher perceived perceived stress was significantly associated with greater internalizing symptoms, including depression, anxiety, and somatization. Family functioning partially mediated this relationship, indicating that prolonged perceived stress adversely affected mental health both directly and indirectly through disruptions in family processes. Qualitative findings corroborated the quantitative results, revealing themes of spatial claustrophobia, erosion of family boundaries, and prolonged uncertainty (“waiting disease”) associated with long-term shelter residence.

**Conclusions:**

Prolonged residence in temporary shelters is associated with substantial psychological distress among earthquake survivors, with family functioning representing a key pathway through which perceived stress impacts mental health. These findings underscore the importance of family-centered psychosocial interventions and improved living conditions in long-term disaster recovery efforts.

## Introduction

On February 6, 2023, a series of powerful earthquakes [Mw 7.8 and Mw 7.5, (1)] struck southeast Türkiye (the Kahramanmaraş earthquakes), displacing 14 million people (2). Even two years later, hundreds of thousands of survivors remain in temporary “container city” settlements, awaiting permanent housing solutions (3). Prolonged displacement on this scale is associated with significant mental health risks (4). Large epidemiological studies indicate that individuals displaced for extended periods after natural disasters show markedly elevated rates of depression and anxiety compared to those not displaced (5). For example, one survey found that survivors displaced for more than six months had nearly *double* the odds of depression and anxiety symptoms relative to non-displaced peers (6). In similar post-earthquake scenarios, persistent residence in temporary camps correlates with high levels of psychological distress (7); nearly two years after the 2008 Wenchuan earthquake in China, over one-third of camp-dwelling survivors exhibited clinical depression (36%) and anxiety (38%), (8). These findings underscore that as the “temporary” phase of disaster recovery drags on, what was initially acute trauma can evolve into chronic psychological strain (9). In addition to anxiety and depression, survivors often report somatic symptoms (e.g., headaches, fatigue, bodily pains) that accompany their emotional distress (10). Such somatization is a well-documented component of post-disaster mental health problems, potentially reflecting the physical manifestation of prolonged stress (11). Together, these outcomes – depression, anxiety, and somatic complaints – represent the internalizing symptom spectrum that is of concern in protracted displacement contexts (12).

Disasters impact not only individual survivors but also the family unit and its functioning. In the aftermath of catastrophe, families can be a critical source of emotional support and resilience (13). Close-knit family bonds and “huddling” together after a disaster have been associated with better coping and recovery (14). However, when displacement is prolonged, the family system itself may come under severe stress (15). The living conditions in high-density temporary settlements – typically small prefabricated container homes (~21 square meters per family) – create daily challenges such as overcrowding and loss of privacy (16). Overcrowding and lack of privacy have been consistently linked to strained family relationships and diminished wellbeing (17). Research on post-disaster and refugee households has observed that when families are confined to inadequate living spaces or face long-term uncertainty, traditional family roles and communication patterns are disrupted (18). For instance, chronic displacement stressors like *loss of housing, economic hardship, and ambiguous waiting periods* often elevate interpersonal tensions (19), sometimes leading to increased family conflict or even domestic violence (20). In humanitarian settings, the uncertainty about the future and frustration of “living in limbo” can manifest as impatience or despair within families. Over time, a once-supportive family may struggle to maintain healthy functioning under these conditions. Boundaries between parent and child roles can blur, privacy for marital or individual respite is scant, and minor disagreements can be amplified by the constant crowding (21). In summary, family functioning in a disaster context can be a double-edged sword: strong family cohesion can buffer stress, but unhealthy family dynamics (brought on or worsened by the displacement environment) may compound each member's psychological burden.

Despite extensive literature on disaster mental health, there is a relative gap in understanding the “chronic phase” of displacement – that is, the psychological and familial impacts on survivors who remain in temporary shelters years after the initial event (22). Most studies of post-disaster mental health focus on the acute aftermath or on populations that have largely returned to permanent homes. Far fewer have examined those who continue to live in provisional accommodations for prolonged periods (one notable exception being studies of protracted refugee camp situations). In Türkiye's case, as of February 2025, roughly *651,958 individuals* were still residing in over 300 container city sites across the affected region (i.e., a scenario of “prolonged transience” on a massive scale). This context raises new questions: How does living for ~24–36 months in a 21 m^2^ container affect one's mental health? Does the strain of long-term temporary living create a unique pattern of distress beyond the initial trauma? And critically, what role do family processes play in survivors' adaptation (or maladaptation) during this protracted waiting period for permanent housing?

The present study was designed to address these questions by examining the interplay of perceived stress, family functioning, and internalizing psychological outcomes among earthquake survivors in the chronic displacement phase. Perceived stress refers here to the perceived stress arising from the post-disaster living environment – including crowding, infrastructure shortcomings, social tensions in the camp, and the general unpredictability of life in a temporary shelter. Family functioning encompasses the health of family relationships and dynamics (e.g., communication, support, conflict resolution) as measured by a standard family assessment instrument. The psychological outcomes of interest are internalizing symptoms: depression, anxiety, and somatization (assessed via the Brief Symptom Inventory subscales). Grounded in family stress theory (23) and ecological frameworks, we posited that the stress of the environment would adversely affect mental health both directly and indirectly through its impact on the family. In other words, stressors related to container city conditions may correspond with greater family dysfunction, which may statistically account for part of the association between perceived stress and internalizing symptoms.

In summary, this study aimed to examine whether family functioning mediates the relationship between prolonged displacement–related stress and psychological distress among earthquake survivors. Based on prior literature and our conceptual model, we expected that higher perceived perceived stress in the temporary housing context would be associated with greater internalizing symptoms, and that this association would be explained, at least in part, by disruptions in family functioning (i.e., survivors reporting poorer family functioning would exhibit higher levels of psychological distress). To address these aims, we employed a convergent mixed-methods design, quantitatively testing a mediation model linking perceived stress, family functioning, and internalizing symptoms, while qualitatively exploring survivors' lived experiences of prolonged shelter residence—such as spatial claustrophobia and boundary dissolution within 21-square-meter living environments. By integrating these complementary approaches, the present study seeks to clarify the family-level pathways through which prolonged displacement contributes to long-term psychological distress in post-earthquake contexts.

## Methods

### Study design

The study employed a convergent parallel mixed-methods design to evaluate the psychological state of earthquake survivors in the chronic phase of displacement—approximately two years after the February 6, 2023 Kahramanmaraş earthquakes. This design involved the concurrent collection and analysis of both quantitative and qualitative data, enabling a multi-layered understanding of how prolonged residence in temporary shelters impacts mental health. The quantitative component was a cross-sectional survey of 413 adults recruited through purposive sampling from several high-density “container city” settlements. Participants were recruited from several high-density temporary shelter settlements (container city sites) across the Kahramanmaraş, Adiyaman, and Hatay provinces, established following the February 2023 earthquakes to house displaced survivors. These sites were chosen as they represent the largest remaining hubs of the 651,958 displaced individuals still awaiting permanent housing as of June 2025. In the study's conceptual framework, perceived stress (operationalized as perceived stress regarding the shelter's physical and social conditions) was the independent variable, and internalizing symptoms (depression, anxiety, and somatization) was the dependent variable. Family functioning was included in the model as a mediating variable to examine whether perceived stress influences psychological outcomes indirectly through family processes. Meanwhile, the qualitative component consisted of semi-structured interviews designed to capture the lived experience of “prolonged transience.” These interviews probed issues such as household boundary violations and the psychological toll of uncertainty while waiting for permanent housing. All data were collected between June and September 2025—a critical window just before the government's 2026 target date for decommissioning the remaining container city settlements.

### Participants

The study focused on the most densely populated temporary housing areas in Kahramanmaraş, Adiyaman, and Hatay—regions that remained the epicenter of displacement two years post-earthquake. As of June 2025, approximately 651,958 people were still living in 397 container city settlements across these provinces while awaiting permanent housing. From these settings, a total of 413 adult earthquake survivors were recruited for the quantitative survey. Key recruitment sites included Baykar and Sakarya Container Cities in Kahramanmaraş, the K-8 and K-11 temporary housing areas in Adiyaman, and the Qatar–Turkey and Kocaeli Brotherhood container settlements in Hatay. After the survey phase, a purposive sub-sample of 41 participants from the original 413 was invited to take part in the qualitative phase. This smaller group was selected using a maximum variation sampling strategy to capture diverse experiences across different ages, genders, and family sizes—thereby enriching the interpretation of the quantitative trends.

Participants were recruited using a direct door-to-door approach by trained research assistants, who visited individual container units in the selected settlements. This face-to-face recruitment was deemed necessary due to limited internet access and the crowded living conditions, which made digital or remote outreach impractical. Inclusion criteria required that individuals meet all of the following: be at least 18 years old, have been directly affected by the February 6, 2023 earthquakes, and have lived in a container city for at least 12 consecutive months by the time of data collection. The 12-month residency criterion was used to ensure participants experienced “prolonged” displacement (operationalized as at least one year in a temporary shelter as of mid-2025). Additionally, because family functioning was a key variable, only individuals living with at least one other family member were eligible to participate.

Exclusion criteria were applied to protect data quality and participant wellbeing. Specifically, individuals were not enrolled if they: had severe cognitive impairment, active psychosis, or a neurological disorder that could impede informed consent or reliable self-reporting; or had already transitioned to permanent housing (e.g., moving into a TOKI apartment or a private rental).

In addition, any returned survey with more than 10% missing data was discarded, yielding a final valid quantitative sample of 413. All qualitative interviews were conducted one-on-one in private locations at the container sites to ensure confidentiality. The study received ethical approval from the Nişantaşi University Institutional Review Board, and all participants (*N* = 413 quantitative; *n* = 41 qualitative) provided informed consent. With respect to sample adequacy, the quantitative sample size (*N* = 413) exceeds commonly recommended thresholds for mediation analysis using regression-based approaches. Simulation studies suggest that samples above 200 participants generally provide stable parameter estimation and adequate power to detect medium indirect effects when using bootstrapping procedures. Although the sample size was determined pragmatically based on field access and feasibility within container settlements, it is sufficient for the mediation model tested in the present study and supports the stability of the reported indirect effects.

### Measures

#### Brief symptom inventory (BSI)

Participants' general psychological distress was assessed with the Brief Symptom Inventory (BSI), a standardized questionnaire developed by Derogatis (24) to measure a broad range of psychiatric symptoms. The BSI has demonstrated high internal consistency in its original form (Cronbach's α = 0.71–0.85 across subscales). Sahin and Durak's (25) Turkish adaptation of the BSI showed excellent reliability, with α = 0.94 for the total score and α = 0.71–0.94 for the subscales. In the present study, the BSI was used to index *internalizing symptoms* (specifically the depression, anxiety, and somatization subscale scores).

#### Perceived stress scale (PSS-10)

Perceived stress was measured with the 10-item Perceived Stress Scale (PSS-10), which assesses the degree to which individuals appraise their life situations as unpredictable, overwhelming, or uncontrollable. The PSS measures general perceived stress rather than context-specific environmental stressors. In the present study, PSS scores were interpreted as reflecting participants' subjective stress appraisal within the context of prolonged shelter residence. The original development by Cohen, Kamarck, and Mermelstein (26) reported internal consistencies of α = 0.84–0.86. In a Turkish validation study, Eskin et al. (27) found a Cronbach's α of 0.82 for the 10-item version. Use of the PSS-10 was considered appropriate given the study context of prolonged displacement in 2025, as it captures the stress of life in unpredictable temporary shelter conditions (e.g., in Baykar and Sakarya container cities).

#### Family assessment device (FAD)

Family functioning was assessed using the General Functioning subscale of the Family Assessment Device (FAD), grounded in the McMaster Model of Family Functioning (28). The General Functioning subscale consists of 12 items and provides a global index of overall family health and dysfunction. The Turkish adaptation by Bulut (29) reported variable reliability across FAD subscales (α = 0.38–0.86), whereas the General Functioning subscale demonstrated stronger psychometric performance (α = 0.90). In the present sample, the General Functioning subscale showed high internal consistency (Cronbach's α = 0.90), supporting its adequacy as a global indicator of family functioning in this study.

### Sociodemographic and contextual questionnaire

Participants also completed a brief questionnaire covering demographic information and displacement-related contextual factors. This form (developed by the researchers) gathered basic demographics such as age, gender, marital status, education level, and household size (number of people, including children, living in the same 21-square-meter unit). In light of the protracted disaster timeline (data collection occurring in late 2025, over two years post-earthquake), the form also asked about the total duration of residence in temporary housing, current employment status, and any economic changes since the earthquakes. Finally, respondents indicated whether they had received permanent housing (e.g., a TOKI home) or were still awaiting resettlement. These contextual variables were recorded to help interpret participants' stress levels and mental health symptoms against the broader socio-economic backdrop of ongoing displacement (with 651,958 people still living in 397 container cities as of late 2025).

### Qualitative interview protocol

The qualitative phase employed a semi-structured interview guide created by the authors to delve into participants' lived experiences. The interview questions were designed to probe three focal areas reflecting the study's main constructs: (1) perceptions of living in a confined 21-square-meter space, (2) shifts in family roles and dynamics over the two-year displacement period, and (3) the emotional toll of “prolonged transience” associated with waiting for permanent housing. To ensure content validity and cultural sensitivity, the interview protocol was reviewed by three experts in clinical psychology and disaster response, and it was piloted with two survivors for feedback.

All 41 qualitative interviews were conducted one-on-one in private spaces within the container city sites to encourage openness and confidentiality. Each interview lasted approximately 45–60 min. This qualitative approach enabled a nuanced exploration of stressors unique to high-density temporary settlements such as Baykar and Sakarya (Kahramanmaraş) and K-8/K-11 (Adiyaman). Topics like spatial constraints and loss of privacy were examined in relation to their impact on family communication and individual wellbeing. The qualitative findings thus provided rich, contextual evidence of the “21-square-meter syndrome” phenomenon, complementing the quantitative results and highlighting the sustained uncertainty faced by those still living in temporary shelters as the 2026 container city closure deadline approached.

### Data analysis

#### Quantitative analysis

All survey data (*N* = 413) were analyzed using IBM SPSS Statistics (Version 29.0). Initially, descriptive statistics (means, standard deviations, and distribution indices like skewness and kurtosis) were computed to summarize the sample's demographics and key study variables. The reliability (internal consistency) of each psychometric scale in this sample was checked by calculating Cronbach's alpha for the BSI, PSS-10, and FAD. Next, to examine the study's hypotheses, a mediation analysis was conducted with Hayes' PROCESS macro (Version 4.2, Model 4). Specifically, we tested whether family functioning mediates the effect of perceived stress on internalizing symptoms. A bootstrapping procedure (5,000 resamples) was used to construct 95% confidence intervals for the indirect effect. This approach allowed identification of significant pathways in the mediation model linking shelter stress to mental health outcomes.

#### Qualitative analysis

Interview data from the 41 participants were analyzed using Braun and Clarke's six-phase thematic analysis procedure. Interviews were audio-recorded and transcribed verbatim, then read multiple times to ensure familiarization with the content. Using NVivo software, authors coded the transcripts and collaboratively developed a set of themes and sub-themes emerging from the data. To enhance rigor, an independent coder double-checked the theme coding, and any discrepancies were resolved through discussion and consensus (inter-coder reliability check). This qualitative analysis captured nuanced experiences such as feelings of spatial claustrophobia and disruptions in family roles (the “21-square-meter syndrome”) reported in late 2025.

#### Mixed-methods integration

Following the convergent parallel design, quantitative and qualitative results were integrated to enrich interpretation. A joint display technique was used to compare statistical findings from the surveys with thematic insights from the interviews side-by-side. The qualitative narratives were examined to explain the “how” and “why” behind key quantitative relationships—for example, elaborating how elevated family dysfunction scores corresponded to observed communication breakdowns in crowded living conditions. By merging these data streams, the study ensured that broad statistical patterns were contextualized with real-life experiences, providing a holistic understanding of the psychological toll on displaced survivors (still numbering around 651,958 as of late 2025).

## Results

### Sample characteristics

The characteristics of the participant samples are summarized in Table 1. In the quantitative survey sample (*N* = 413), participants were on average 38.6 years old (SD = 11.4). The gender distribution was roughly equal (54.2% female; 45.8% male). A large majority (82.8%) of respondents were married and living with their families in the temporary shelters. Educational backgrounds were diverse, though most participants had completed high school. The mean household size was about 4.2 people, and on average participants had spent 29.2 months (approximately 2.4 years) living in a container shelter as of the survey. Additionally, nearly two-thirds (62.5%) of the sample were unemployed or reliant on social assistance during the study period. The qualitative sub-sample (*n* = 41) had a very similar profile. Their mean age was 40.2 years (SD = 9.8), with 56.1% female, and 85.4% married; household size was comparable (around 4.4 persons per container). These 41 individuals were selected to provide depth to the statistical findings. The qualitative interviews were conducted in mid-2025, aiming to capture survivors' transition from the initial post-disaster period to the later “chronic” phase of displacement. Notably, this period coincided with growing delays in permanent housing delivery, marking a shift from early optimism to frustration as government housing projects fell behind schedule.

**Table 1:** Characteristics of the study sample.

Variable	Quantitative (*N* = 413)	Qualitative (*n* = 41)
Gender (female/male)	224 (54.2%)/189 (45.8%)	23 (56.1%)/18 (43.9%)
Age (Mean ± SD)	38.6 ± 11.4 years	40.2 ± 9.8 years
Marital status (Married)	342 (82.8%)	35 (85.4%)
Employment status (unemployed/assistance)	258 (62.5%)	27 (65.8%)
Household size (mean)	4.2 individuals	4.4 individuals
Duration of shelter stay (mean)	29.2 ± 1.4 months	29.5 ± 1.2 months
Location: Kahramanmaraş	165 (40.0%)	16 (39.0%)
Location: Adiyaman	128 (31.0%)	13 (31.7%)
Location: Hatay	120 (29.0%)	12 (29.3%)

### Descriptive statistics

**Figure 1:**
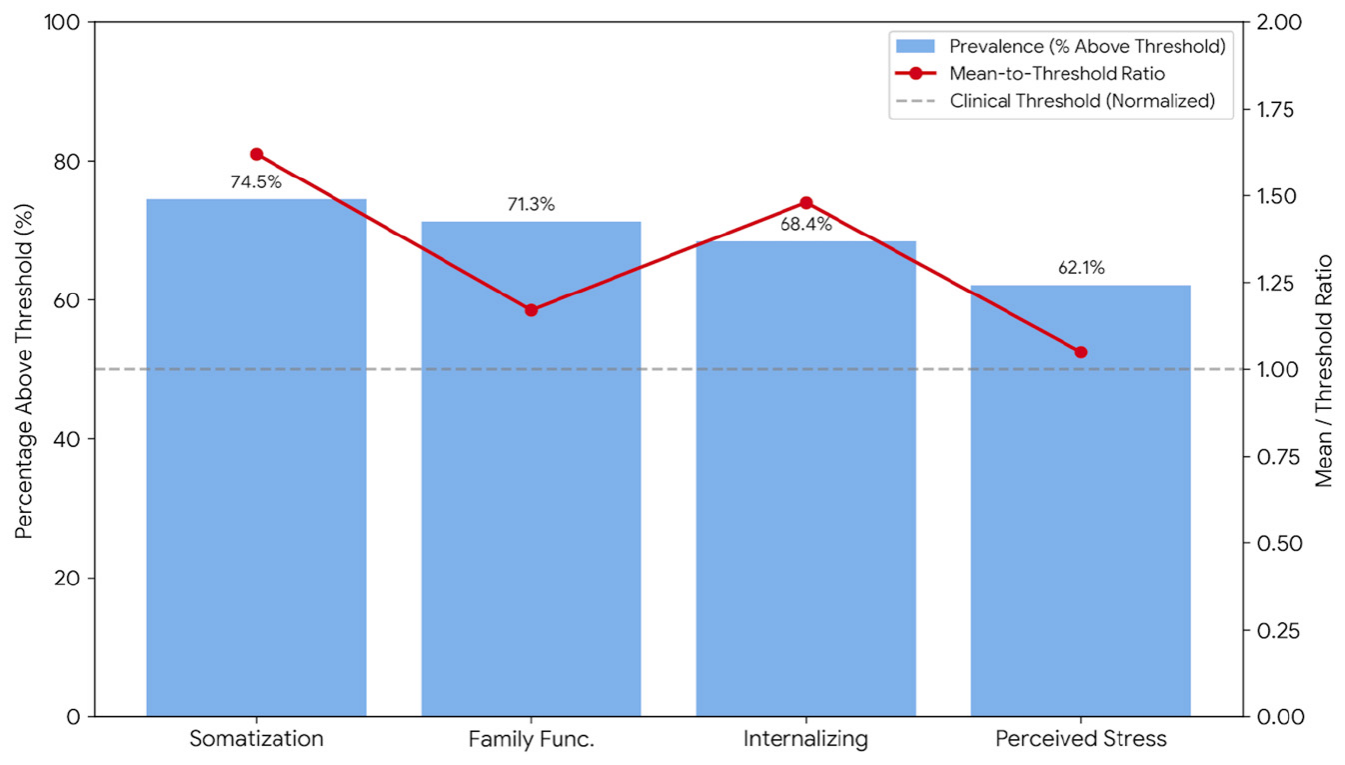
Clinical severity and prevalence of psychological distress (*N* = 413).

Summary statistics for the major psychological measures are presented in Table 2. All scales showed good internal consistency in the current sample (Cronbach's α = 0.82 for the PSS-10; 0.90 for the FAD general functioning; and 0.94 for the BSI total score). On average, participants reported elevated internalizing symptoms (BSI Global Severity Index mean = 1.48, SD = 0.65), well above the general Turkish normative mean of approximately 1.00. The somatization subscale was especially elevated (mean = 1.62, SD = 0.72), suggesting that the prolonged displacement has begun to manifest in chronic physical complaints. Perceived stress levels were also high (PSS-10 mean = 27.2, SD = 5.8), placing most individuals in a high-stress category. In parallel, the mean family functioning score (FAD General Functioning = 2.34, SD = 0.42) exceeded the threshold of 2.00 that denotes unhealthy family dynamics. This indicates that the family unit—typically a source of resilience—had been strained by the crowded living conditions. Indeed, as shown in Figure 1, approximately 71.3% of households in the survey fell into the “unhealthy” range on family functioning, illustrating the widespread erosion of effective family roles and communication after over two years of communal living in temporary shelters.

**Table 2:** Psychometric properties and descriptive statistics of the study scales (*N* = 413).

Variable	Number of items	Observed range	Mean	SD	Cronbach's alpha
Internalizing symptoms (BSI index)	53	0.12–3.84	1.48	0.65	0.94
Somatization (BSI subscale)	7	0.00–4.00	1.62	0.72	0.88
Perceived stress (PSS-10)	10	12–40	27.2	5.8	0.82
Family functioning (FAD general)	12	1.15–3.75	2.34	0.42	0.90

The clinical profile of the 413 participants reveals a population in a state of chronic somatic and social exhaustion two and a half years after the disaster. As illustrated in the clinical severity chart (Figure 1), Somatization yielded the highest elevation (Mean = 1.62), indicating that the psychological toll of residing in a 21-square-meter unit for an average of 29.2 months has transitioned into physical distress. Furthermore, 71.3% of households scored in the unhealthy range for family functioning, suggesting that the spatial constraints of the container cities have systematically undermined family functioning as a source of psychological support.

### Bivariate correlations

Pearson correlation coefficients among the key variables are presented in Table 3. The analysis showed that higher perceived perceived stress was moderately associated with higher internalizing symptom levels (r = 0.56, *p* < 0.01). Family dysfunction (higher FAD scores) was also significantly correlated with greater internalizing symptoms (r = 0.48, *p* < 0.01). In addition, perceived stress was positively correlated with unhealthy family functioning (r = 0.42, *p* < 0.01), indicating that more stressful living conditions were linked to more impaired family relationships. Notably, perceived stress had a substantial correlation with somatization severity (r = 0.51, *p* < 0.01), reinforcing the observation that the “waiting phase” of prolonged displacement is not only psychologically taxing but also linked to physical symptomatology in survivors.

**Table 3:** Intercorrelations between primary variables.

Variable	1	2	3	4
1. Perceived stress (PSS-10)	—			
2. Family functioning (FAD - General Functioning)	0.42^**^	—		
3. Internalizing symptoms (BSI)	0.56^**^	0.48^**^	—	
4. Somatization (BSI Subscale)	0.51^**^	0.39^**^	0.78^**^	—
^*^p < 0.01				

^*^p < 0.05; ^**^p < 0.01; ^***^p < 0.001.

### Mediation analysis

**Figure 2:**
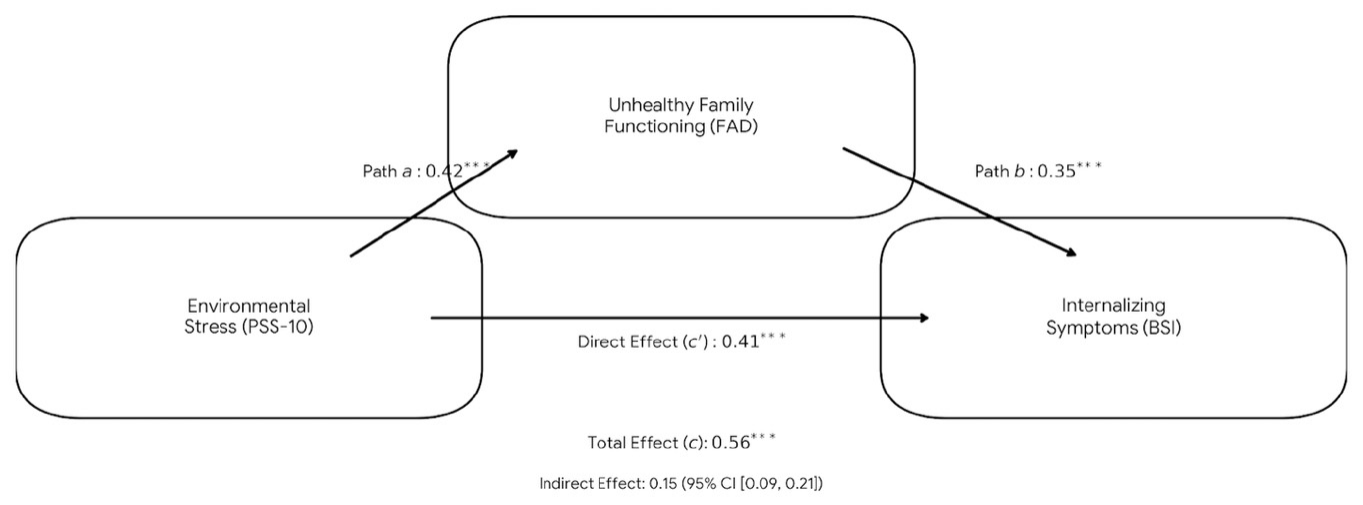
Mediation analysis of environmental stress on mental health through family functioning.

A mediation analysis (PROCESS Model 4) was performed to examine whether family functioning accounted for (mediated) the relationship between perceived stress and internalizing symptoms. Table 4 summarizes the regression coefficients for this model. The findings supported a partial mediation effect. Specifically, higher perceived stress predicted poorer family functioning (Path a: β = 0.42, SE = 0.04, *t* = 10.50, *p* < 0.001). In turn, poorer family functioning significantly predicted higher internalizing symptom levels when controlling for stress (Path b: β = 0.35, SE = 0.05, *t* = 7.00, *p* < 0.001). The total effect of perceived stress on internalizing symptoms was significant (Path c: β = 0.56, SE = 0.06, *t* = 9.33, *p* < 0.001). Importantly, the direct effect of perceived stress on symptoms remained significant but was reduced after accounting for family functioning (Path c′: β = 0.41, SE = 0.05, *t* = 8.20, *p* < 0.001). The indirect effect via family functioning was estimated at β = 0.15, and its 95% bootstrap confidence interval [0.09, 0.21] did not include zero. Thus, as illustrated in Figure 2, higher perceived stress was associated with poorer family functioning, which in turn was associated with higher internalizing symptoms. These findings are consistent with a model in which family functioning statistically accounts for part of the association between stress and psychological distress.

**Table 4:** Mediation analysis results for the structural model (*N* = 413).

Path Relationship	Beta	SE	*t*-value	*p*-value	95% CI (LL, UL)
Path a: stress to family functioning	0.42	0.04	10.50	< 0.001	(0.34, 0.50)
Path b: family functioning to symptoms	0.35	0.05	7.00	< 0.001	(0.25, 0.45)
Path c: total effect (Stress to symptoms)	0.56	0.06	9.33	< 0.001	(0.44, 0.68)
Path c': direct effect (Stress to symptoms)	0.41	0.05	8.20	< 0.001	(0.31, 0.51)
Indirect effect: (a × b)	0.15	0.03	—	—	(0.09, 0.21)

In Figure 2, the results indicate that environmental stress significantly predicts a deterioration in family functioning (Path a: beta = 0.42, *p* < 0.001), which in turn serves as a significant predictor of internalizing symptoms (Path b: beta = 0.35, *p* < 0.001). While the direct effect of perceived stress on symptoms remained significant (Path c': beta = 0.41, *p* < 0.001), the identification of a significant indirect effect [0.15, 95% Confidence Interval (0.09, 0.21)] confirms a partial mediation. This pattern is consistent with what participants described as the “21-square-meter syndrome”: the physical constraints of high-density sites like Baykar and Sakarya are associated with higher levels of individual distress and correspond to indicators of strained family functioning.This erosion of the family buffer subsequently amplifies the psychological burden on survivors during the chronic and ongoing “waiting phase” identified in late 2025.

### Qualitative results

Qualitative analysis of the interviews (*n* = 41) yielded several recurring themes, summarized in Table 5 along with their definitions and representative quotes. Three major themes emerged, each aligning with patterns observed in the quantitative data: (1) *Spatial Claustrophobia*, (2) *Boundary Dissolution and Erosion of Family Roles*, and (3) *Prolonged Transience* (often referred to by participants as the “Waiting Disease”). The following sections describe each theme in detail.

**Table 5:** Thematic framework of the psychological experiences of earthquake survivors (*n* = 41).

Main theme	Definition	Representative participant quote
Spatial claustrophobia (21 sqm Syndrome)	The suffocating sensation caused by restricted living space and its physical (somatic) manifestations.	“The four walls feel like they are closing in on me... 21 square meters is no longer a home; it feels like a box where we cannot breathe.”
Boundary dissolution and erosion of family roles	The breakdown of family hierarchy and communication quality due to the absolute lack of privacy.	“Sleeping in the same room with children, arguing in the same space... I can no longer tell if I am a mother or a wife; we have no boundaries left.”
Prolonged transience and the “Waiting Disease”	The sense of being stuck between the uncertainty of permanent housing and the 2026 decommissioning targets.	“Our lives are on hold. We are being exhausted while waiting for the TOKI; not knowing when it will end is more tiring than the earthquake itself.”

#### Spatial claustrophobia (“21-square-meter syndrome”)

Nearly all interviewees described intense feelings of confinement associated with living long-term in a 21 m^2^ container. This *spatial claustrophobia* was repeatedly linked to physical symptoms, mirroring the elevated somatization levels in the survey results. Survivors explained that what was initially a temporary shelter had, after about 30 months, come to feel like a restrictive “cage” affecting both mind and body. As one participant described, “21 square meters is no longer a home; it feels like a box where we cannot breathe.” Many residents reported chronic headaches, sleep disturbances, and other somatic complaints that they attributed to the cramped, inescapable living environment. These accounts are consistent with the possibility that prolonged spatial constraints correspond to reductions in perceived coping capacity and to elevated somatic symptom reporting.

#### Boundary dissolution and erosion of family roles

The second major theme was the breakdown of family boundaries and roles due to the complete lack of privacy in the shelters. This theme provides context to the quantitative finding that 71.3% of surveyed households had unhealthy family functioning. Interviewees emphasized that living, sleeping, and carrying out all daily activities in a single shared room had essentially *dissolved* normal boundaries between family members. One mother explained, “Sleeping in the same room with the children, arguing in the same space... I can no longer tell if I am a mother or a wife; we have no boundaries left.” With no private space for anyone, traditional family hierarchies and parenting practices collapsed. Participants noted that even minor disagreements quickly escalated in the crowded setting, and the family unit had lost its role as an emotional safe haven. In essence, the container housing environment itself had become a chronic stressor that was undermining the family system from within.

#### Prolonged transience (the “Waiting Disease”)

The final theme encapsulated the chronic uncertainty and sense of powerlessness among survivors stuck in limbo. By mid-2025, delays in the rollout of permanent TOKI housing—along with the looming 2026 deadline to close all container cities—had created what participants grimly referred to as a “waiting disease.” People no longer saw themselves simply as disaster victims but as *permanent* victims of transience. One interviewee lamented, “Our lives are on hold. We are exhausted from waiting for the TOKI; not knowing when it will end is more tiring than the earthquake itself.” This liminal state corresponded with the high perceived stress scores observed quantitatively. Participants conveyed that genuine recovery felt impossible without some control over their future. In other words, improving mental health would require not only the delivery of permanent housing but also the restoration of personal agency after more than two years of dislocation.

## Discussion

The findings of this study largely support the proposed model and hypotheses, revealing how deeply the prolonged displacement experience has affected survivors' mental health and family life (30, 31). First, as hypothesized, we found that perceived perceived stress in the container city was strongly associated with internalizing psychological symptoms. Survivors who reported higher stress related to their living environment (e.g., feeling confined, lacking control or safety, enduring the chaos of camp life) also reported significantly higher levels of depression, anxiety, and somatization. This aligns with prior research showing that the longer people remain displaced after a disaster, the greater their risk for mental health problems (32, 33). The strength of this association in our data echoes patterns observed in other post-disaster contexts. For instance, in the Wenchuan earthquake sample discussed earlier, survivors still in temporary camps two years post-quake exhibited substantially elevated rates of depression and anxiety compared to those who had relocated (34). Our results reinforce this concerning trend: the chronic stress of living in limbo correlates with a chronic psychological toll. Many participants in our study have been living in container shelters for ~30 months – a period long enough for initial acute stress reactions to potentially evolve into more entrenched conditions like depressive and anxiety disorders. The fact that somatic complaints were also high among those under greater stress suggests that survivors are not only mentally but also physically *feeling* the strain of prolonged displacement [consistent with evidence that disaster-related distress often manifests in bodily symptoms (35)]. Importantly, given the cross-sectional nature of the data, the observed mediation pattern should be interpreted as statistical rather than causal. Although the model was theoretically informed, the temporal ordering among perceived stress, family functioning, and internalizing symptoms cannot be established. It is equally plausible that individuals experiencing higher levels of depression or anxiety perceive their living conditions as more stressful or report poorer family functioning. Bidirectional and transactional processes are likely operating in prolonged displacement contexts.

Crucially, our study goes beyond documenting this stress-distress link by illuminating the role of family functioning as both an outcome of stress and a predictor of mental health. As expected, we found that worse (unhealthier) family functioning was significantly correlated with higher internalizing symptoms (36). Survivors who described their families as having poor communication, low cohesion, high conflict, or role breakdown tended to score higher on depression, anxiety, and somatization scales (37). This finding resonates with a large body of literature showing that when family relationships deteriorate, individual mental health often suffers – whether in everyday contexts or in high-stress situations like displacement (38). More specifically, our results indicated that families living under the extreme conditions of the container city were, in many cases, not able to maintain a supportive, adaptive environment, and this in turn left individuals more vulnerable to psychological distress. This dynamic is consistent with family stress models [e.g., Conger's Family Stress Model, (39)] and ecological theories, which propose that external stressors [such as economic hardship or, in our case, disaster-induced homelessness, (40)] can disrupt family processes – leading to marital strain, impaired parenting, or family conflict – which then negatively affect individual wellbeing. Our findings are consistent with this theoretical framework in a disaster context: the “21-square-meter syndrome” described by participants reflects an perceived stress context that is associated with poorer family functioning and elevated mental health symptoms (41).

The mediation analysis (see Table 4) provided more formal support for this interpretation. It showed that family functioning partially mediates the effect of perceived stress on internalizing symptoms. In the mediation model, higher perceived stress significantly predicted more dysfunctional family functioning, which in turn predicted higher depression, anxiety, and somatization scores. Notably, even after accounting for family functioning, perceived stress still had a direct effect on psychological symptoms (i.e., partial mediation rather than full mediation). This pattern is consistent with two statistically distinguishable associations: a direct association between difficult living conditions and internalizing symptoms, and an indirect association through family functioning. The existence of the indirect pathway confirms our hypothesis that the family acts as a conduit for some of the stress. Qualitatively, this was reflected in participants' descriptions of fraying family bonds – for example, parents feeling they could not fulfill their roles properly, increased arguments over space and privacy, or feelings of guilt and frustration circulating among family members – all of which contributed to individuals' emotional distress. These findings are consistent with prior observations that displacement-related stressors, such as crowded living conditions and prolonged uncertainty, are associated with strained family dynamics, including increased conflict or harsh parenting practices. Our quantitative results align with this literature by indicating that family functioning statistically accounts for part of the association between perceived stress and internalizing symptoms. However, several alternative explanations warrant consideration. First, pre-disaster family dynamics may have shaped how families adapted to displacement; families with pre-existing vulnerabilities may have experienced greater strain in the container settlements. Second, pre-existing mental health conditions could influence both perceived perceived stress and reported family functioning. Third, reciprocal processes are plausible: psychological distress may contribute to interpersonal tension, which in turn may reinforce stress perceptions. These possibilities underscore the importance of viewing the observed associations within a transactional framework rather than as evidence of unidirectional effects. From a transactional or ecological systems perspective (23, 40), stress and family functioning may influence one another over time, suggesting a dynamic interplay rather than a fixed directional pathway.

It is also worth noting that the mediation was partial, indicating that family factors, while important, do not entirely explain the link between environment and mental health. Even in families that managed to function relatively well, the sheer adversity of the prolonged displacement had a residual impact on survivors' mental health. This implies that family functioning, while relevant, appeared insufficient to fully offset the effects of prolonged perceived stress– a nuance that aligns with our expectations. We hypothesized that healthy family functioning might buffer individuals from the full impact of perceived stress (i.e., a moderating effect). However, moderation was not statistically supported in the present analyses. Future longitudinal and intervention studies are needed to directly test whether family functioning operates as a protective factor under conditions of prolonged displacement. In essence, our findings suggest that “family matters” – variations in family functioning were associated with differences in psychological symptom severity under conditions of prolonged displacement. This interpretation is bolstered by broader disaster research showing that survivors who feel supported by family fare better, and that family breakdown or violence post-disaster is a major risk factor for mental illness. Thus, our results integrate with existing literature by highlighting family functioning as a key intermediate variable linking disaster *environmental* conditions to *individual* outcomes.

### Mixed-methods integration and mechanistic interpretation

The integration of quantitative and qualitative findings extends beyond simple confirmation and provides additional contextual and theoretical nuance. First, the quantitative association between perceived stress and somatization was illuminated by the qualitative theme of *spatial claustrophobia*. Participants' descriptions of physical confinement, chronic headaches, sleep disruption, and bodily tension suggest that somatic symptoms may represent embodied stress responses to prolonged environmental constraint. From an ecological stress perspective, sustained exposure to spatial restriction may function as a chronic stress context that shapes both psychological and physiological symptom expression.

Second, the mediating role of family functioning in the statistical model was enriched by the qualitative theme of *boundary dissolution and erosion of family roles*. While the quantitative data indicated that family functioning statistically accounted for part of the association between perceived stress and internalizing symptoms, interview narratives revealed how this process may unfold in daily life—through blurred parental roles, loss of privacy, increased interpersonal friction, and role strain. These accounts align with family stress theory by illustrating how external stressors become internalized within family systems, potentially disrupting communication patterns and role organization.

Third, the theme of *prolonged transience* (“waiting disease”) provided contextual depth to the elevated perceived stress scores observed in the survey data. Participants emphasized uncertainty, loss of control, and suspended life trajectories as central sources of distress. This suggests that perceived stress may reflect not only immediate environmental strain but also anticipatory uncertainty and diminished agency, consistent with ecological models of chronic stress exposure.

Importantly, the qualitative findings also suggest additional processes not directly tested in the quantitative model. Economic strain, caregiving burden, gendered role expectations, and loss of occupational identity emerged as salient stressors that may function as potential mediators or moderators in future research. For example, women frequently described intensified caregiving demands within confined spaces, while unemployed participants emphasized economic instability as compounding psychological strain. These themes indicate that prolonged displacement likely operates through multiple intersecting pathways beyond those modeled statistically in the present study.

### Implications for disaster mental health interventions and policy

These findings carry several important implications for intervention and policy in disaster contexts. First and foremost, mental health interventions for displaced survivors should adopt a family-centered approach. Traditional post-disaster mental health efforts often focus on individuals (for example, providing counseling for a survivor with PTSD or medication for depression). While such individual treatment is valuable, our results indicate it may be insufficient if the survivor returns each night to a highly stressful home environment in the camps. It is crucial to also address the family system that surrounds the individual. Practically, this could mean providing family-based psychosocial support – for instance, family therapy sessions in the camp, parenting workshops, or facilitated support groups where multiple family members participate together. There is evidence from refugee settings that family-based mental health interventions (such as multi-family discussion groups or parenting skills programs) can improve both family functioning and individual outcomes. By strengthening communication, problem-solving, and emotional support within families, such interventions could help break the mediated chain we observed (whereby perceived stress → family dysfunction → individual distress). In a disaster-relief scenario, implementing family interventions might involve training non-specialist counselors or community volunteers to deliver basic family counseling or conflict resolution training, given that resources are limited. Importantly, leveraging family strengths and resilience may extend the reach of mental health efforts, as families with healthier functioning are often associated with more sustained psychological support beyond formal therapy sessions.

Secondly, our study underscores the need to improve living conditions and reduce chronic stressors in temporary settlements as a matter of public policy. The fact that perceived stress had a direct impact on mental health means that no amount of counseling or family work can fully compensate for a highly adverse environment. Policymakers and disaster response authorities should aim to make container cities and other temporary housing as livable and dignified as possible. This includes addressing issues like privacy and crowding. For example, simple infrastructure modifications – providing partitions or screens to partition space within a container, creating separate areas for different families or for different activities (sleeping vs. living areas), or establishing community facilities (bathhouses, kitchens, social spaces) to relieve the pressure on each small unit – could mitigate some daily stress. Overcrowding and lack of privacy are not just inconveniences; they are risk factors for psychological distress. Humanitarian guidelines already acknowledge this, recommending that post-disaster shelters be designed to support safety and privacy as much as possible. Our findings put empirical weight behind those recommendations. Additionally, camp management could implement schedules or rules to reduce chaotic aspects of daily life – for example, structured distribution of resources to avoid long stressful queues, quiet hours to allow rest in the crowded camp, and private counseling or *safe spaces* where individuals can briefly escape the family gaze when needed. Such environmental and administrative improvements, while not a substitute for permanent housing, could help alleviate the chronic stress that currently permeates survivors' lives.

Another key policy implication is the urgency of expediting permanent housing and “closing the limbo” of displacement. Two years on, tens of thousands of Turkish survivors are still in container camps facing an uncertain timeline for rehousing. Our qualitative findings vividly illustrate a phenomenon that some participants called “*the waiting disease”* – a sense of stagnation, hopelessness, and loss of agency stemming from the protracted wait for normal life to resume. This resonates with reports from humanitarian organizations that the psychological burden of uncertainty remains heavy among survivors even two years post-disaster. From a policy perspective, this means that reconstruction timelines and resettlement plans should be viewed through a mental health lens, not just an economic or logistical one. Delays in providing permanent housing are not merely inconveniences; they correspond with continued psychological strain and reduced adaptive capacity among displaced families. Therefore, accelerating construction and repair of homes (where feasible) is a mental health intervention in its own right. In Türkiye's case, continuing to invest in rapid building of safe permanent residences or expanding programs that subsidize moves to existing housing could alleviate the “permanent transient” status that so many families endure.

Of course, large-scale housing projects take time. In the interim, policy efforts should also focus on providing robust psychosocial support and socioeconomic opportunities to those stuck in temporary settlements. Mental Health and Psychosocial Support (MHPSS) services need to be consistently available on-site – such as clinics or mobile teams offering counseling, psychiatric care, and community-based activities that restore a sense of normalcy (schooling for children, community events, etc.). The Turkish Red Crescent and other agencies have indeed been offering mental health services in the camps, but coverage can always be expanded given the level of need. Alongside direct mental health services, economic and livelihood support is crucial. Many survivors in our study mentioned unemployment and financial strain as exacerbating their stress. Programs like cash assistance, job training, and small business grants can help families regain stability and autonomy. Evidence from the earthquake response shows that cash aid has been vital in restoring a sense of control and reducing anxiety for families who lost their livelihoods. By improving a family's financial security, such support can indirectly reduce intra-family tension (fewer arguments about scarce resources) and improve mental health (through increased hope and self-efficacy). In summary, an integrated approach is needed: one that combines expediting physical reconstruction, enhancing camp living conditions, delivering family-oriented mental health interventions, and supporting socio-economic recovery. These combined efforts would address both the underlying stressors (the challenging environment and prolonged uncertainty) and the psychological and relational difficulties identified in our study.

### Limitations

While this study provides valuable insights, several limitations must be acknowledged. First, the design was cross-sectional, meaning all data on stress, family functioning, and mental health were collected at the same single time point (approximately two years post-disaster). This limits our ability to draw causal conclusions. We have interpreted the results in line with our conceptual model (assuming, for instance, that perceived stress led to family dysfunction, which then led to distress), but it is possible that the relationships are bidirectional or influenced by prior conditions. For example, individuals with pre-existing mental health issues might perceive their environment as more stressful, or families that were dysfunctional even before the earthquake might both struggle more in the camp and have members with higher symptom levels. Longitudinal research – tracking survivors from initial displacement through various time points – would help clarify the directionality of these effects. Furthermore, cultural context may shape both stress appraisal and family functioning processes. The present sample consisted predominantly of Turkish families residing within structured container settlements; patterns of family dynamics and stress perception may differ in societies with alternative kinship structures or social safety nets. Similarly, the findings may not generalize to other disaster types (e.g., floods, armed conflict, pandemics), where displacement trajectories and stressors differ qualitatively. Finally, the results are specific to high-density container housing environments and may not apply to individuals residing in tents, informal settlements, or host-family arrangements, where privacy, autonomy, and social integration dynamics may vary substantially.

An additional consideration concerns potential selection bias. Participants were recruited through door-to-door visits and voluntary participation within container settlements. Individuals experiencing severe psychological distress may have been less likely to participate due to fatigue, stigma, or withdrawal, whereas those with strong opinions about living conditions may have been more motivated to engage. Consequently, the sample may not fully represent all displaced residents, particularly the most vulnerable or socially isolated individuals. These recruitment dynamics should be considered when interpreting prevalence levels and effect sizes.

Second, the study relied on self-report measures for key variables. Perceived stress, family functioning, and psychological symptoms were all reported by participants themselves (via questionnaires like the Perceived Stress Scale, Family Assessment Device, and Brief Symptom Inventory). Self-report can introduce biases such as social desirability (e.g., a participant underreporting family problems to appear more “normal”) or common method variance that might inflate correlations. We attempted to mitigate these issues by assuring participants of confidentiality and using well-validated instruments. Nonetheless, future studies could complement self-reports with objective or multi-informant data – for instance, observations of family interactions, clinical interviews for mental health diagnoses, or reports from multiple family members (to see if, say, a parent and teen agree on their family's level of functioning).

An additional limitation concerns the use of the FAD General Functioning subscale as a global composite measure. While this subscale provides a reliable index of overall family health, it does not capture the full multidimensional structure of family functioning, such as communication patterns, role organization, affective responsiveness, and behavioral control. Future research would benefit from examining specific FAD subdomains to better understand which aspects of family dynamics are most strongly associated with perceived stress and psychological distress in prolonged displacement contexts.

Third, the generalizability of our findings may be limited by the specific sample and context. We focused on survivors in high-density container cities in three hard-hit provinces (Kahramanmaraş, Adiyaman, and Hatay). These sites had certain characteristics – for example, they were relatively organized camps with container units, and residents had survived a major earthquake and remained displaced due to slow reconstruction. The experiences of these participants might differ from survivors in other contexts: those living in informal or rural temporary shelters (like tents or with host families) might report different stressors; survivors of other disasters (such as floods or conflicts) might have additional trauma dynamics at play; and cultural factors in this predominantly Turkish sample (e.g., strong extended family networks) might influence how displacement affects family life. Caution is warranted in extending the conclusions to all disaster-displaced populations. That said, the core issues we identified – environmental hardship, family strain, and mental distress – are common themes in disaster recovery literature, suggesting a degree of transferability to analogous situations.

An additional limitation concerns construct specificity. PSS-10 assesses general perceived stress rather than environment-specific or shelter-related stressors. Although the scale was administered within the context of prolonged container city residence, it does not directly measure objective housing quality, crowding indices, or environmental infrastructure conditions. Therefore, findings should be interpreted as reflecting subjective stress appraisal rather than the direct effects of physical environmental characteristics. Future research would benefit from incorporating context-specific housing quality or environmental stress measures alongside general perceived stress instruments.

Finally, our study's scope was somewhat narrow in terms of the psychological outcomes examined. We concentrated on internalizing symptoms (depression, anxiety, somatization) as indicators of distress. We did not formally assess post-traumatic stress disorder (PTSD) or other trauma-specific symptoms, even though many participants undoubtedly experienced trauma from the earthquake and aftershocks. PTSD could be a significant outcome in this population and may interact with family functioning in complex ways (for instance, a family member's PTSD symptoms could strain family relationships, or vice versa). Additionally, we did not examine externalizing or behavioral problems (such as aggression or substance abuse) which can also increase in post-disaster settings and affect family dynamics (for example, substance use could both be a coping mechanism for stress and a source of family conflict). Our qualitative data hinted at some anger and frustration issues, but these were not quantified. Future research should broaden the range of outcomes to provide a more holistic picture of mental health in prolonged displacement. Despite these limitations, the study offers a valuable snapshot of a critical phase in disaster recovery, and the mixed-methods approach strengthens confidence in the patterns observed by cross-validating them with survivors' personal narratives.

### Recommendations for future research

Building on this study's insights, we can outline several directions for future research. First, as noted, longitudinal studies are needed to track how mental health and family functioning evolve over time in displaced populations. Following families from the initial emergency (e.g., within weeks of the earthquake) through the chronic displacement phase, and then into the resettlement phase (after moving to permanent housing), would be immensely informative. Such research could answer questions like: Does mental health improve spontaneously once families are rehoused, or do some issues persist? Which is more long-lasting – the direct impact of the traumatic event, or the wear-and-tear of two years spent in temporary living? And does family functioning rebound once stressors are alleviated, or do rifts that formed in the camps carry forward? Longitudinal data could also help identify causal pathways – for example, using cross-lagged panel models to see if earlier family problems predict later psychological symptoms, or vice versa.

Second, future research should consider interventional and experimental designs to test some of the implications we have discussed. For instance, one could design a study to evaluate a family-strengthening intervention in a disaster camp – perhaps randomly assigning some displaced families to receive a structured family therapy program (or a community-based family support program) while others receive standard care, and then measuring differences in family functioning and mental health outcomes over time. If our interpretation is correct that improving family functioning can buffer individuals from stress, such an intervention should yield better psychological outcomes compared to a control condition. Similarly, intervention studies could test environmental modifications: for example, providing one group of camp residents with improved housing amenities or privacy solutions and comparing their stress and mental health outcomes to those in standard containers. Ethically and logistically, these trials can be challenging in disaster settings, but even pilot programs or quasi-experiments could yield valuable evidence on what supports are most effective.

Third, researchers should delve deeper into the mechanisms and additional mediators that link prolonged displacement to mental health. Our study highlighted family functioning as a key mediator, but there are likely other important factors. Social support networks beyond the family (friends in the camp, relationships with aid workers) might mitigate distress. Sense of control or agency is another candidate – some survivors may cope better if they find ways to exert control (such as organizing camp activities or advocating for their needs), whereas feeling helpless could worsen mental health. Meaning-making and cultural beliefs might also play a role; for example, survivors who ascribe religious or spiritual meaning to the disaster might experience different trajectories of coping. Qualitative data from our and others' work suggest that maintaining hope and purpose is critical during the “waiting” period. Future studies could use mixed-methods designs to identify such themes and then quantitatively measure their impact. By expanding the lens to these additional factors, we can construct a more comprehensive theoretical model of “prolonged disaster resilience (or pathology)” that includes individual, family, and community-level processes.

Additionally, it would be worthwhile to investigate the intergenerational aspects of prolonged displacement. Our study focused on adult survivors, but many families included children and adolescents. Children growing up in a container city for two years have had their formative experiences in an abnormal environment – crowded living, possibly interrupted schooling, and parents under great stress. Research could examine outcomes for youth (emotional, behavioral, educational) and how they relate to family functioning and parental mental health. There is evidence from other contexts that children's wellbeing after disasters is closely tied to how well their parents cope and how stable the family unit remains. Conversely, children's distress can feed back into parental stress. Studying these family system dynamics with a focus on children could inform interventions (for example, family-based interventions that also incorporate child-focused trauma therapy or play therapy). Moreover, as these displaced children age, longitudinal research could explore if prolonged displacement has any lasting effects on their development or if they show resilience once normalcy returns.

## Conclusion

Overall, this study provides a comprehensive look at the psychological and familial impact of prolonged disaster displacement in the context of the 2023 Türkiye earthquakes. Two years into the recovery, survivors remaining in temporary container camps exhibited high levels of depression, anxiety, and somatic complaints, highlighting an ongoing mental health crisis. Our findings demonstrate that the stress of living in limbo – in crowded, impermanent conditions – is taking a measurable toll on survivors' wellbeing, and that this toll is partly channeled through the breakdown of family functioning. The assumption that family functioning consistently operates as a protective factor was challenged, as prolonged displacement appeared to undermine family processes themselves. Yet, instances of resilience were also evident: not all families disintegrated, and those that maintained healthier dynamics tended to have members with better mental health. These nuances underscore that effective disaster mental health responses must be multi-faceted. Interventions should support individuals *and* families, and policy efforts must reduce environmental stressors *and* expedite a return to stability. In practical terms, this might mean providing counseling that involves family units, creating safer and more private living spaces in camps, offering clear timelines and communication about housing plans, and ensuring access to economic opportunities and community support. As the disaster literature moves beyond the immediate aftermath and examines the long tail of recovery, studies like ours reinforce a crucial insight: “Disasters don't end when the shaking stops or the waters recede.” For displaced survivors, the disaster's effects often continue in the form of daily stress, uncertainty, and altered family life. Mental health professionals, relief organizations, and policymakers should anticipate these chronic effects and address them with the same urgency as acute trauma. The findings presented here, we hope, contribute to a growing evidence base that can inform better practices – so that in future disasters, families like those in Kahramanmaraş, Adiyaman, and Hatay receive not only emergency aid in the first weeks, but also sustained support in the months and years that follow. By recognizing the fundamental human needs for stability, home, and family cohesion, we can shape interventions that help survivors reclaim their mental wellbeing and rebuild their lives even before the bricks-and-mortar reconstruction is complete.

## Data Availability

The original contributions presented in the study are included in the article/supplementary material, further inquiries can be directed to the corresponding author.
